# Management of Squamous Cell Carcinomas of the Anal Canal and Anal Margin After Failure of Chemoradiotherapy Treatment: A Narrative Review

**DOI:** 10.3390/cancers17091511

**Published:** 2025-04-30

**Authors:** Michaël Racine, Guillaume Meurette, Frédéric Ris, Jeremy Meyer, Christian Toso, Emilie Liot

**Affiliations:** Visceral Surgery, Department of Surgery, Geneva University Hospital, 1205 Geneva, Switzerland

**Keywords:** anal squamous cell carcinoma, treatment, recurrence, persistence

## Abstract

Anal squamous cell carcinoma (ASCC) is a rare malignancy with an increasing incidence despite advancements in treatment. The primary treatment for localized ASCC is radiochemotherapy (RCT), which achieves high rates of tumor regression in most cases, but up to 30% of patients experience recurrence or persistent disease. Salvage surgery, such as an abdominoperineal resection (APR), is often used for recurrent disease but is associated with significant morbidity and limited oncological outcomes. Patients with small T1 tumors may also benefit from primary local excision. For patients with metastatic or unresectable recurrent ASCC, chemotherapy, particularly carboplatin and paclitaxel, remains the standard treatment. New therapeutic strategies, including immune checkpoint inhibitors like pembrolizumab, are showing promise, particularly in PD-L1-positive tumors. Clinical trials have suggested that immunotherapy offers a potential alternative for patients for whom conventional treatments have failed, though the overall response rates remain modest. Re-radiation and intraoperative radiotherapy combined with salvage surgery may improve the outcomes for select patients, though the data are still limited. The management of recurrent or persistent ASCC requires a personalized approach, incorporating both established and emerging therapies to optimize patient outcomes. Further research is needed to refine these treatment strategies.

## Simple Summary

Anal squamous cell carcinoma (ASCC) presents a challenge in clinical practice due to its rarity and its multifaceted management. This comprehensive narrative review focuses more particularly on therapeutic strategies in cases of recurrence or persistence following the initial therapy. Drawing insights from recent studies, this review explores the role of salvage abdominoperineal resection (APR), chemotherapy, radiotherapy, and immunotherapy for optimizing outcomes. By synthesizing the current evidence and discussing emerging trends, this review aims to provide an understanding of the evolving strategies for managing ASCC.

## 1. Introduction

Anal cancer is a rare malignancy accounting for a small percentage (<3%) of gastrointestinal cancers [1]. The anal canal stretches from the anorectal junction to the anal margin. The anal margin refers to the pigmented skin surrounding the anal orifice, extending laterally to a radius of approximately 5 cm [1]. Cancer occurring within the anal margin is considered anal cancer, while cancer located more than 5 cm away from the anus is classified as skin cancer [1]. Anal squamous cell carcinoma (ASCC) is the most common histological subtype. Despite advancements in treatment modalities, the prevalence of ASCC has been on the rise in the United States, Western Europe, Australia, and South America [2], necessitating a deeper exploration of therapeutic approaches. The major risk factor is HPV infection, accounting for 80–85% of cases [3], with HPV 16 being the most often seen. So, sexual behavior and immunosuppression are the key elements [3]. Primary radiochemotherapy (RCT) remains the cornerstone of ASCC treatment. This approach has been shown to achieve high rates of tumor regression and loco-regional control, leading to complete tumor regression in 80–90% of patients. The actual recommendations are based on multiples trials (phase II and six randomized phase III trials) [4,5,6,7,8,9,10]. Concomitant 5-Fluorouracyl (5-FU) and Mitomycin C with radiation therapy is generally recommended. Other options may include 5-FU and cisplatin. The only exceptions are for small tumors of the perianal skin or anal margin not involving the anal sphincter that may be adequately treated with only a local resection. Nevertheless, the recurrence rate or non-response after the initial chemoradiation therapy can vary. Studies have reported that up to 30% of patients may experience treatment failure, leading to either persistent disease or local recurrence [11]. Salvage surgery plays a crucial role in cases of disease recurrence or persistence post-radiation [12]. In addition to salvage surgery, emerging treatment modalities, such as immunotherapy and targeted therapies, have shown promise in the management of ASCC. Immunotherapy, including checkpoint inhibitors, has revolutionized the treatment landscape of various cancers [13,14,15]. Studies have explored the efficacy of immunotherapy in ASCC, with some trials showing encouraging results [16]. Targeted therapies against specific pathways, such as the TGF-β pathway, are also being investigated in ASCC [16]. These novel approaches offer potential alternatives for patients who are not candidates for or for whom conventional treatments have failed. This review aims to provide an in-depth analysis of the management of ASCC, focusing on the therapeutic strategies for scenarios of disease recurrence or persistence after initial radiation.

## 2. Management Strategies in Local/Loco-Regional Disease

### 2.1. Salvage Surgery

Salvage surgery plays a crucial role in the management of anal squamous cell carcinoma (ASCC) by offering a treatment option for patients with recurrent or persistent disease following initial RCT [17]. In cases of a primary resection for small T1 tumors, RCT is still a possible treatment for recurrence or persistence. It is essential to closely monitor patients post-chemoradiation therapy to assess their treatment response. The majority of tumors that persist or recur typically do so within the first 24 months following the completion of chemoradiotherapy (CRT). A non-response at 3 months does not indicate the need for salvage treatment, since reassessment at 6 months often shows clinically significant regression [18]. Salvage surgery, such as an extended abdominoperineal resection (APR), is often a difficult and potentially morbid surgery with significant lifestyle implications. A study evaluating the quality of life in disease-free survivors following salvage surgery for ASCC found that while the overall quality of life was acceptable, there were notable side effects impacting the patients’ well-being [19]. Impotence, dyspareunia, and urinary impairment were reported as common side effects [19]. In certain cases, APR, involving the removal of the rectum and anus, is insufficient. A pelvic exenteration may then be required, during which the following organs may be resected, depending on the extent of the cancer and its involvement: bladder, uterus, vagina, cervix, prostate, and even sacrum. In certain cases, additional structures may be removed, including nearby lymph nodes and parts of the pelvic wall, if there is extensive disease spread [20].

While primary closure (PC) has traditionally been a common practice, the rate of perineal wound complications varies between 25 and 60% [21]. A key factor contributing to these complications is the significant dead space that remains in the pelvic cavity following an oncological resection. To reduce the likelihood of perineal complications, alternative closure techniques are necessary, particularly since most patients will have undergone chemoradiotherapy. Abdominal-based flaps, such as a vertical rectus abdominis myocutaneous (VRAM), transverse rectus abdominis myocutaneous (TRAM), or deep inferior epigastric artery perforator (DIEP) flaps, have been used [21]. These flaps generally benefit from a non-irradiated blood supply, although they may lead to longer surgical times. Alternatively, thigh-based flaps, including gracilis, anterolateral, and gluteal flaps, are effective options, particularly for smaller wounds. A myocutaneous flap reconstruction of the perineum, like a VRAM flap, has proven to be a valuable alternative to primary closure [21]. The rationale behind flap reconstruction is that it not only addresses the pelvic dead space but also enhances wound healing and lowers the risk of infection due to its robust blood supply [21]. VRAM flaps help lower the incidence of wound complications, shorten the healing time, and decrease the likelihood of perineal herniation, all without raising the risk of developing abdominal incisional hernias [22]. In the systematic review and meta-analysis of Temperley et al. from 2024 [21], 38.2% (263/688) of patients in the primary closure group versus 32.8% (80/244) in the VRAM group showed perineal wound complications. A meta-analysis using the M-H random-effects model revealed a significant difference in perineal complication rates between the two groups, with a significantly lower rate in the VRAM group (M-H OR, 1.61; 95% CI, 1.04–2.49; *p* = 0.03). Concerning perineal wound dehiscence, no significant difference was shown between the two groups (25.4% for primary closure and 28.2% for VRAM) [21].

Recent findings from Kitaguchi et al. from 2023 [12] highlighted the importance of considering the type of disease (recurrent vs. persistent) when evaluating the effectiveness of salvage APR in patients with anal SCC. Indeed, survival outcomes following salvage APR for patients with recurrent disease were significantly better than for those with persistent disease. There was a significantly positive effect of a salvage APR on the overall survival (OS) for patients with recurrent disease (5-year overall survival 75% vs. 42% without salvage APR), but not for those with persistent disease (5-year overall survival 36% vs. 47% without salvage APR). The study suggests that there was no additional benefit of a salvage APR for the persistent disease group.

In another recent study by Rosen et al., published in 2024 [23], they presented their work as “among the largest single institutional studies to present the morbidity, oncological, and survival outcomes after a salvage APR for anal SCC in patients who underwent combined modality treatment at a comprehensive cancer center”, and showed that a salvage APR has poor oncologic outcomes, with a 3-year disease-specific survival, post-APR local recurrence-free survival, and disease-free survival of 53.8% (95% CI, 43.5 to 66.5%), 54.5% (95% CI, 44.4 to 66.8%), and 26.8% (95% CI, 18.6 to 38.7%), respectively. Unlike the Kitaguchi et al. study [12], the indication for an APR (recurrent vs. persistent) did not influence the survival outcomes. The risk factors for recurrence and decreased survival after salvage surgery include positive resection margins, positive lymph nodes, and the presence of lymphovascular invasion in the resected specimen.

Hagemans et al. [24] published, in 2018, their results for 47 consecutive salvage APRs for persistent or recurrent ASSC over 30 years. The median overall survival (OS) was 47 months, with a 5-year survival rate of 41.6%, which did not significantly differ between the patients with persistent or recurrent disease. Twenty-one patients developed a local recurrence after a salvage APR. Increased pathological tumor size and lymph node involvement were associated with an impaired hazard for overall survival in the multivariable analysis. As in other studies, an APR was a morbid operation, with perineal wound complications reported in 31.9% of the patients (perineal hernia, wound debridement, and the need for muscle flap reconstruction). Accounting for all the complications (Dindo–Clavien 1–5), only 36.2% of the patients had none.

A systematic review of outcomes after a salvage APR for persistent or recurrent ASSC published in 2019 by Ko et al. [11], including 28 retrospective case series, showed that the median time to a salvage APR was 2.6 months for persistent disease and 27.6 months for recurrent disease. The median 5-year overall survival rates following salvage APRs were 45.0% for persistent disease and 51.0% for recurrent disease. The weighted, median 5-year disease-free survival (DFS) following a salvage APR was 44.0% for all the patients. The median loco-regional recurrence rate was 23.5%, and 9.0% of the patients developed metastatic disease. The study did not specifically mention the complications following surgery.

Fields et al. [25] published, in 2019, a study on a total of 437 patients with nonmetastatic ASSC who underwent a salvage APR. The study found that there was no significant difference in the overall survival between the patients who underwent an early salvage abdominoperineal resection (APR) within 6 months of completing chemoradiotherapy and those who had a late salvage APR 6 months or more after the completion of chemoradiotherapy. The overall survival of the patients in the early salvage group was 48.4%, while in the late salvage group, it was 40.3%.

The following table summarizes the results of the various studies mentioned above (Table 1).

In most cases, the inguinal nodes should be included in the radiotherapy fields during the primary treatment, even if there is no clear evidence of involvement [1]. The likelihood of inguinal nodal involvement increases with the size of the primary tumor and is at least 20% in patients with T3 disease [1]. As a corollary, the persistence or recurrence of disease in inguinal lymph nodes is rare (<5%). Moreover, surgery at this level is morbid due to prior irradiation [1]. A paper from Lin et al. from 2020 showed no differences in the 5 y overall survival between an APR alone or an APR with an inguinal lymphadenectomy [26]. Given the limited evidence available, clear guidelines are difficult to implement. The ESMO, for example, states that in these situations, “surgery should be considered”, but that a “less radical lymph node excision might be appropriate”.

In short, a salvage APR is a procedure fraught with significant morbidity for modest oncological results. New treatment modalities are therefore desirable.

### 2.2. Chemotherapy

For the overall 10–20% of patients who experience recurrent metastatic disease, systemic therapy is the mainstay of treatment [1]. Moreover, of the 20% of patients who will show recurrence or non-response after primary CRT, only a certain percentage will be eligible for salvage surgery. For the remainder, systemic treatment is indicated [1].

Currently, a combination of carboplatin and paclitaxel remains the standard treatment in these situations [1,2]. This regimen has shown an overall survival of 20 months compared to 12.3 months for the cisplatin—5-FU combination in the largest and only multicenter, randomized, controlled phase 2 study (The International Multicentre Study in Advanced Anal Cancer—InterAACT) [27]. Kim et al. reported, in 2018 [28], their results concerning patients with metastatic or unresectable recurrent ASSC treated with docetaxel, cisplatin, and fluorouracil (DCF) in the standard form (75 mg/m^2^ docetaxel and 75 mg/m^2^ cisplatin on day 1 and 750 mg/m^2^ per day of fluorouracil for 5 days, every 3 weeks) or modified form (40 mg/m^2^ docetaxel and 40 mg/m^2^ cisplatin on day 1 and 1200 mg/m^2^ per day of fluorouracil for 2 days, every 2 weeks). In total, 31 (47%) of the 66 patients were alive and progression-free at 12 months. For those on the standard DCF regimen, 22 (61%) of 36 patients experienced disease progression at the data cutoff, while 18 (60%) of 30 patients on the modified DCF regimen had disease progression. The median progression-free survival for the overall population was 11.0 months (10.7 months for the standard DCF regimen, 11 months for the modified DCF regimen). The overall survival at 12 months for the overall population was 83.1%. The survival rates were similar for both regimens: 83.3% for the standard DCF group and 82.7% for the modified DCF group. The median overall survival was not determined for either regimen. Out of the 66 patients, 46 (70%) experienced at least one grade 3–4 adverse event. The most common adverse events included neutropenia, diarrhea, asthenia, and anemia. The modified DCF regimen had fewer severe adverse events compared to the standard DCF regimen and was associated with better tolerability.

In the context of local recurrence, the role of pre- or post-salvage surgery chemotherapy is not known and the data are lacking.

### 2.3. Radiotherapy

Given the relatively high recurrence rates after salvage surgery alone, research has been conducted on the use of intraoperative radiotherapy (IORT) and/or external beam re-radiotherapy (reRT) in addition to surgery.

A research study by Damron et al. from 2024 [29] compared the outcomes of patients who underwent salvage surgery alone versus those who received a multidisciplinary salvage treatment involving reirradiation (reRT) and/or intraoperative radiation. The factors associated with the risk of a second local recurrence and overall survival were analyzed. The study aimed to identify the high-risk groups that could benefit from escalated salvage treatment. The research was conducted between 2002 and 2022 and included a total of 64 patients with locally recurrent SCCA after definitive CRT. The 5-year overall survival rate was 63% for the patients treated with surgery alone and 89% for those who underwent reRT followed by surgery. The 5-year cumulative incidence of a second local recurrence was 45% after salvage surgery alone and 15% after reRT followed by surgery. However, there were significant differences in the patient and tumor characteristics between the surgery-alone and the reRT-followed-by-surgery groups, and the multivariable analysis did not show improved pelvic control or an overall survival benefit in the reRT or IORT groups at the time of surgery. Nevertheless, the patients with positive surgical margins and lymphovascular space invasion on the surgical pathology had higher rates of pelvic recurrence after salvage surgery and may benefit from escalated salvage therapy.

Hallemeier et al. [30] published, in 2014, a study including 32 patients. In the patients with residual disease after primary CRT, immediate salvage surgery and IORT were performed without the administration of additional preoperative CRT. In the patients with recurrent disease, preoperative low-dose external beam radiation therapy (eBRt) was administered. All the patients underwent a salvage surgical resection. The 5-year estimates for overall and disease-free survival were 23% and 17%. The 5-year estimates for central, local–regional, and distant failure were 21%, 51%, and 40%. The rate of treatment-related morbidity was as high as 47% of the grade 3 complications, which was not higher than the rates in the patients undergoing salvage APRs without additional radiotherapy.

Proton therapy appears to be a promising option in cases of anal cancer recurrence where surgery is not feasible, as it allows for better preservation of the healthy tissues (bladder, intestines, skin, and genital organs) and a potential reduction in side effects compared to conventional radiotherapy, while still delivering an effective dose to the recurrent tumor. Although the data remain limited, several centers have published case series or retrospective studies. A 2020 review from Vaios et al. demonstrated that proton therapy used for reirradiation could offer good local tumor control with acceptable toxicity [31].

In short, there are currently insufficient data on radiotherapy treatment combined with salvage surgery. In the latest ESMO guidelines (2021), surgery remains the only salvage treatment at present.

### 2.4. Immunotherapy

The main advancement in cancer treatment over the past decade has been the introduction of T cell-targeted immunomodulators that block immune checkpoints CTLA-4 and PD1 or PDL1 that are expressed by tumor cells and permits them to avoid T cells’ antitumor activity. In 2011, ipilimumab, the first antibody for blocking the CTLA-4 checkpoint, was approved. This was quickly followed by the development of monoclonal antibodies targeting PD1 (pembrolizumab and nivolumab) and PDL1 (atezolizumab and durvalumab). These T cell-targeted immunomodulators are now used for approximately 50 cancer types [32].

Concerning ASCC, the study by Ott et al. from 2017 [33] contributed to the understanding of treatment options for recurrent carcinoma of the anal canal by evaluating the efficacy and safety of pembrolizumab, an anti-PD-1 antibody, in a cohort of patients with PD-L1-positive advanced anal carcinoma. They reported an overall response rate of 17% in patients with squamous cell carcinoma histology, indicating that a subset of patients may benefit from pembrolizumab after the failure of prior standard therapies. This was significant, as it represented one of the first published results on immune checkpoint blockade in this specific cancer type, providing a potential new treatment avenue for patients with limited options after conventional therapies failed. The study found a high rate of PD-L1 positivity (74% of screened patients) in anal carcinoma, suggesting that PD-L1 expression may be a relevant biomarker for selecting patients who could benefit from a PD-1 blockade. This finding aligns with the understanding that PD-L1 expression is associated with a higher antitumor activity of PD-1 inhibitors in other tumor types, thus reinforcing the rationale for using pembrolizumab in this context. The study also highlighted the manageable safety profile of pembrolizumab in this patient population.

Marabelle et al. [34] reported, in 2022, on the efficacy and safety of pembrolizumab in patients with already-treated advanced ASSC, irrespective of their PD-L1 status, from the phase II KEYNOTE-158 study. KEYNOTE-158 was an international, open-label, non-randomized, multicohort, multicenter, phase 2 study conducted across 38 centers. It evaluated pembrolizumab in patients with various rare advanced solid tumor types, including ASSC. A total of 112 patients were enrolled in the ASSC cohort. A total of 9% of the patients completed the 2-year treatment. The most common reasons for stopping treatment were progression in 82% of the patients and adverse event in 6% of the patients. An objective response (complete and partial response) was achieved in 11% of the patients (15% in the PD-L1-positive group and 3% in the PD-L1-negative group). The median progression-free survival was 2.1 months and the median overall survival was 12.1 months in the patients with PD-L1-positive tumors. In the patients with PD-L1-negative tumors, the median progression-free survival was 2.0 months and the median overall survival was 12.1 months.

Kim et al. [35] published, in 2024, the “SCARCE C17-02 PRODIGE 60” phase 2 trial results evaluating the anti-tumor activity and safety of the combination of atezolizumab immunotherapy and modified DCF (mDCF) chemotherapy for patients with chemo-naive, metastatic, or unresectable locally advanced recurrent squamous cell carcinoma of the anus. The participants were randomly assigned (2:1) to receive either the first-line mDCF and atezolizumab (experimental group A; 64 patients) or mDCF alone (control group B; 33 patients). The 12-month progression-free survival (PFS) was 45% for group A and 43% for group B and, thus, the addition of atezolizumab to the mDCF regimen did not demonstrate a substantial improvement in the overall response rates compared to mDCF alone. A more favorable response to the treatment was observed in a subgroup of patients with a PD-L1 combined positive score (CPS) of 5% or greater. In this subgroup, the treatment was more effective (PFS 70%), suggesting that PD-L1 levels could serve as a potential biomarker for predicting responses to the combination therapy.

The following table summarizes the results of the various studies mentioned above (Table 2).

According to the ESMO guidelines from 2021, checkpoint inhibitors should be considered as an appropriate treatment option for patients with advanced ASCC who have progressed on first-line treatments in clinical trials. The National Comprehensive Cancer Network also recommends anti-PD-1 therapy as the preferred subsequent treatment for patients with metastatic anal cancer who have progressed on first-line chemotherapy.

### 2.5. Cryotherapy

Cryotherapy in the treatment of recurrent anal cancer remains poorly documented and is still considered an experimental option [36]. However, some recent studies have suggested its potential in specific situations. A review published in 2023 in *Frontiers in Oncology* examined the indications and contraindications for cryotherapy for low rectal and anal cancers. It highlighted that cryotherapy may be considered as an alternative or a complement to local resection, particularly in cases of local recurrence following chemotherapy and radiotherapy. It is especially suited for small, well-defined, localized tumors, and can be used for palliative purposes to relieve symptoms or reduce tumor burden [36].

## 3. Discussion

Anal squamous cell carcinoma (ASCC) remains a challenging malignancy with an increasing incidence despite advancements in treatment. The cornerstone of treatment for localized or loco-regional ASCC remains RCT, which is effective in 80–90% of cases [4,5,6,7,8,9,10]. However, up to 30% of patients may experience treatment failure, leading to either persistent disease or recurrence [11], highlighting the need for alternative strategies. Salvage surgery, such as an abdominoperineal resection (APR), is often employed for patients with recurrent disease [17], but its morbidity and limited oncological outcomes make it less than ideal. Studies have shown that survival rates after salvage surgery vary depending on the type of recurrence, with those with recurrent disease having better outcomes than those with persistent disease [12]. Despite this, the procedure is fraught with complications, including high rates of local recurrence [23] and significant lifestyle impacts [19].

In the context of metastatic or recurrent disease not suitable for surgery, chemotherapy remains the main treatment option, with carboplatin and paclitaxel being the standard regimen [1,2]. Data on chemotherapy in combination with salvage surgery are scarce. The role of radiotherapy, specifically re-radiation or intraoperative radiotherapy, is still not well established, although some studies have suggested that re-radiation combined with salvage surgery may reduce local recurrence rates and improve survival in select patients [29,30].

Immunotherapy, particularly immune checkpoint inhibitors targeting PD-1/PD-L1, represents an exciting new avenue for ASCC treatment. Clinical trials, such as KEYNOTE-158, have shown promising results with pembrolizumab in patients with advanced or recurrent ASCC, particularly those with PD-L1-positive tumors [32]. Although the overall response rates are modest, this approach offers an option for patients for whom conventional treatments have failed and may offer a more tolerable treatment regimen compared to chemotherapy [33].

**Figure 1:**
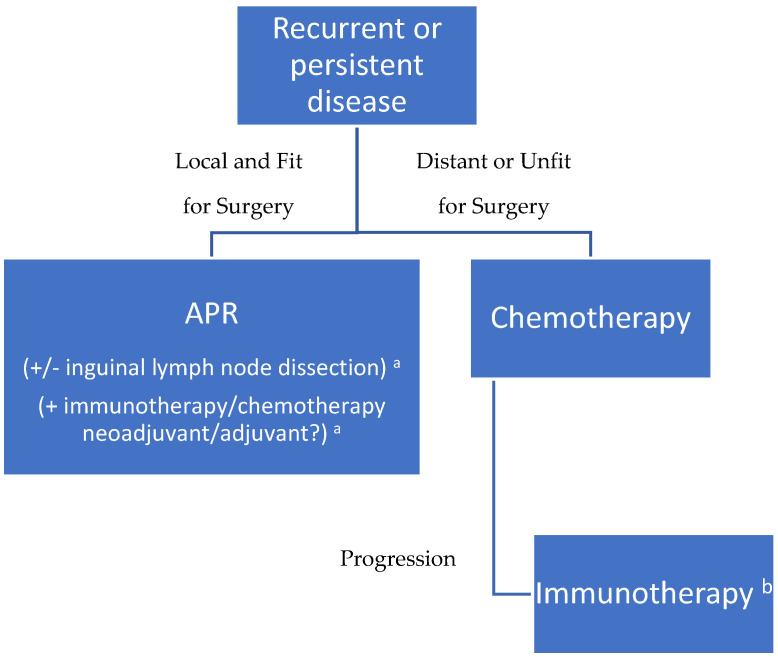
Suggested management flowchart. ^a^ Insufficient evidence; ^b^ as part of clinical trials.

In the following figure, we present a flowchart outlining the management of the disease based on everything that has been discussed (Figure 1).

## 4. Conclusions

The management of recurrent or persistent ASCC remains challenging, with conventional treatments like salvage surgery offering limited success and significant side effects. New therapeutic strategies, including immunotherapy, are emerging as promising alternatives for patients with refractory disease. The use of immune checkpoint inhibitors, particularly pembrolizumab, has shown potential in clinical trials, especially for PD-L1-positive tumors. However, further studies are needed to optimize these treatments and establish clear guidelines for their use. Ultimately, a personalized approach, incorporating both conventional and novel therapies, is essential to improve outcomes for patients with ASCC.

## Figures and Tables

**Table 1:** Summary of salvage surgery studies.

First Author (Year)	Type of Study		Sample Size (Number of Patients)		Median Follow-Up (Months)
Kitaguchi (2023) [12]	Multicentric retrospective cohort study		152		49
Rosen (2024) [23]	Monocentric retrospective cohort study		96		22
Hagemans (2018) [24]	Monocentric retrospective cohort study		47		80
Ko (2019) [11]	Systematic review		1018		NR
Fields (2019) [25]	National Cancer Database (USA) retrospective review		437		27.1 (early salvage); 26.6 (late salvage) ^1^
**First Author (Year)**	**Overall Survival** **(Persistent)**	**Overall Survival (Recurrent)**	**Overall Survival (Overall)**	**Disease-Free** **Survival (Overall)**	**Recurrence-Free** **Survival (Overall)**
Kitaguchi (2023) [12]	36% (5 y)	75% (5 y)	71% (5 y)	NR	64% (5 y)
Rosen (2024) [23]	NR	NR	NR	26.8% (3 y)	54.5% (3 y)
Hagemans (2018) [24]	40.4% (5 y)	41.7% (5 y)	41.6% (5 y)	NR	51.1% (5 y)
Ko (2019) [11]	45% (5 y)	51% (5 y)	NR	44% (5 y)	NR
Fields (2019) [25]	NR	NR	48.4% (5 y, early salvage); 40.3% (5 y, late salvage) ^1^	NR	NR

^1^ Early: within 6 months of completion of CRT; late: >6 months; NR: non recorded.

**Table 2:** Summary of immunotherapy studies.

First Author (Year)	Type of Study	Sample Size (Number of Patients)	Tumor Status	Treatment
Ott (2017) [33]	Multicenter, open-label, phase 1b trial	25	PD-L1 positive	Pembrolizumab
Marabelle (2022) [34]	Non-randomized, multicohort, multicenter, phase 2 study	112	PD-L1 positive or negative	Pembrolizumab
Kim (2024) [35]	Randomized, non-comparative, phase 2 study	97	PD-L1 positive or negative	Pembrolizumab + mDCF
**First Author (Year)**	**Median Progression-Free** **Survival (Months)**	**12-Month Progression-Free Survival Rate**	**Median Overall** **Survival (Months)**	**12-Month Overall** **Survival Rate**
Ott (2017) [33]	3	19.70%	9.3	47.60%
Marabelle (2022) [34]	2	15%	11.9	49%
Kim (2024) [35]	9.4	45%	NR	77%
